# Screening of 20 Commonly Used Iranian Traditional Medicinal Plants Against Urease 

**Published:** 2014

**Authors:** Mahmood Biglar, Hessameddin Sufi, Kowsar Bagherzadeh, Massoud Amanlou, Faraz Mojab

**Affiliations:** a *Department of Medicinal Chemistry, Faculty of Pharmacy and Pharmaceutical Science Research Center, Tehran University of Medical Sciences, Tehran, Iran.*; b *Department of Pharmacognosy, School of Pharmacy, Shahid Beheshti University of Medical Sciences, Tehran, Iran. *

**Keywords:** Urease, *H. pylori*, Medical plants, Extracts, Urease inhibitor

## Abstract

Infection with *Helicobacter pyloriis *the most common cause of stomach and duodenal ulcers. About more than 80 % of people are infected with *H. pylori *in developing countries. *H. pylori *uses urease enzyme product “ammonia” in order to neutralize and protect itself from the stomach acidic condition and urease enzyme activity has been shown to be essential to the colonization of *H. pylori. *Inhibitory activity of 20 traditional medicinal plants were examined and evaluated against Jack bean urease activity by Berthelot reaction to obtains natural sources of urease inhibitors. Each herb was extracted using 80% aqueous methanol, then tested its IC_50_ value was determined. Eight of the whole 20 studied plants crude extracts were found the most effective with IC_50_ values of less than 100 μg/mL including *Laurus nobilis, Zingiber officinale, Nigella sativa, Angelica archangelica, Acorus calamus, Allium sativum,Curcuma longa, and Citrus aurantium *extracts, from which most potent urease inhibitory was observed for *Zingiber officinale, Laurus nobilis*, and *Nigella sativa *with IC_50_ values of 48.54, 48.69 and 59.10 μg/mL, respectively.

## Introduction

Urease (E.C 3.5.1.5), considered as the most proficient protagonist in biochemistry, is a nickel containing enzyme carrying out the rapid catalysis and hydrolysis of urea to produce ammonia and carbon dioxide (1) It diffuses along the cytoplasmic membrane, increases the preplasmic space pH and as a result allows the bacteria growth in the present of extra cellular gastric acid(2). Additionally, urease activity will lead to kidney stones formation and also conduct the development of urolithiasis, pyelonephritis and hepatic encephalopathy (3, 4). 

It has been shown that *H. pylori,*a pathogen which is colonized in the digestion system of human beings and considered as one of the important factors leading to gastric disease,is incapable of causing infection in the absence of urease (5). Natural medicines especially medicinal plants have been considered as one of the options to cure the diseases in some cases for many decades and their basic ingredients are used in medicine industry at present time. Unfortunately along with improvements in discovery of new chemicals, drugs and different antibiotics, not only the harmful side effects of these medicines have emerged, but also the bacteria developed resistance to them. Therefore, discovering of new active chemicals from medicinal plants with possible urease inhibitory activity could help to cure ulcer and gastritis caused by *H. pylori *infection (6, 7). In this study, the urease inhibitory activities of 20 herbal medicine extract against Jack bean urease were evaluated (8,9).

## Experimental

Sodium nitroprussid and urease (EC 3.5.1.5) from Jack beans were purchase from sigma (St. Louis, MO, USA) and deionized water was used in all experiments. Potassium phosphate buffer(100 mM), pH=7.4, was prepared in distilled water. The studied plants were randomly obtained from local medicinal herb shops, Tehran, Iran (June 2012) based on their traditional uses for gastritis and were identified by one of our authors of the presented article (F. Mojab). The authenticated samples were deposited in the Herbarium of Shahid Beheshti University of Medical Science. 

*Extract preparation*

0.5 g of air dried and powdered plant material was extracted in 10 mL, 80:20 methanol: water at room temperature (25±1 ºC) for 24 hours. The resulting liquid extract was then filtered and concentrated to dryness under reduced pressure. The dry extracts were stored at -20 ºC till used (9).

*Determination of urease activity*

All 20 extracts were tested for their urease inhibitory activity at concentration of 125 μg/mL by the modified spectrophometric method developed by Berthelot reaction. Inhibition assays were first performed for herbal extracts that were proven to exert significant inhibition and also for positive controls. The plant extract were tested in a concentration range of 1 to 125 μg/mL. Hydroxyurea was used as standard inhibitor. The solution assay mixture consisted of urea (850 μL) and (135 μL) crud extract with a total value of 985 μL. The reactions were initiated by the addition of 15μL of urease enzyme solution in phosphate buffer (100 mM, pH 7.4, 1 μg/mL). Urease activity was determined by measuring ammonia concentration after 60 minutes of enzymatic reaction. The ammonia was determined using 500 μL of solution A (contained 0.5 g phenol and 2.5 mg of sodium nitroprusside in 50 mL of distilled water) and 500μL of solution B (contained of 250 mg sodium hydroxide and 820 μL of sodium hypochlorite 5% in 50 mL of distilled water) at the temperature of 37 °C for 30 minutes. The absorbance was read at 625 nm. Activity of uninhibited urease was designated as the control activity of 100%.

*Determination of IC*_50_ *values and Data processing*

The extent of the enzymatic reaction was calculated based on the following equation:

*I (%) = 100 – 100* * *(T / C)*

Where *I *(%) is the inhibition of the enzyme, T (test) is the absorbance of the tested sample (plant extract or positive control in the solvent) in the presence of enzyme,C(control) is the absorbance of the solvent in the presence of enzyme. Data are expressed as mean ± standard error (SD) and the results were taken from at least three times.

IC50 values (concentration of test compounds that inhibits the hydrolysis of substrates by 50%) were determined by studying the extracts urease inhibitory activity at their different concentrations in comparison to their individual positive control employing spectrophotometric measurement. IC50 values were obtained from dose-response curves by linear regression, using Graphpad software, prism 5.

## Results and Discussion

In the presented study, urease enzyme inhibition potency of 20 herbal extracts was investigated from which 8 extracts including *Zingiber officinale, Laurus nobilis, Nigella sativa, Angelica archangelica, Acorus calamus, Allium sativum, Curcuma longa, and Citrus aurantium *extracts have shown inhibitory activity with IC_50_ values of less than 500 μg/mL(Table 1).

**Table 1:** Urease inhibitory activity of plants extract

**IC**50 **(μg/mL)**	**Part used**	**Common Name (English)**	**Family**	**Scientific Name**	**No.**
774.82	Flower	Yarrow, Milfoil	Compositae	*Achillea millefolium*	1
88.77	Root	Sweet flag	Araceae	*Acorus calamus*	2
170.42	Root	Garlic	Lilliaceae	*Allium sativum*	3
64.30	Leaf	Garden angelica	Apiaceae	*Angelica archangelica*	4
691.48	Seed	Black Mustard	Brassicaceae	*Brassica nigra*	5
740.11	Bark	Quinine bark tree	Rubiaceae	*Cinchona officinalis*	6
465.24	Peel	Bitter orange	Rutaceae	*Citrus aurantium*	7
310.54	Rhizome	Turmeric	Zingiberaceae	*Curcuma longa*	8
763.23	Seed	Thon – apple	solanaceae	*Daturastramonium*	9
580.17	Seed	Fennel seed	Apiaceae	*Foeniculum vulgare*	10
634.67	Root	Yellow gentian	Gentianaceae	*Gentiana lutea*	11
651.91	Twig	Hops	Cannabinaceae	*Humulus lupulus*	12
703.12	Herb	Hyssop	Labiatae	*Hyssopus officinalis*	13
48.69	Leaf	Bay tree , Laurel tree	Lauraceae	*Laurus nobilis*	14
786.71	Flower	Common Mallow	malvaceae	*Malva sylvestris*	15
59.10	Seed	Black Cumin	RanunCulaceae	*Nigella sativa*	16
603.32	Seed	Black pepper	Piperaceae	*Piper nigrum*	17
725.36	Root	Madder	Rubiaceae	*Rubia tinctorum*	18
523.74	Herb	Fenugreek	Leguminosae	*Trigonella foenum – graceum*	19
48.54	Rhizome- root	Ginger root	Zingiberaceae	*Zingiber officinale*	20

 Further examinations and IC50 determination revealed that the most potent urease inhibitory was observed for *Zingiber officinale *(48.54 μg/mL)*, Laurus nobilis *(48.69 μg/mL), *Nigella sativa *(59.10 μg/mL), *Angelica archangelica *(64.03 μg/mL), and *Acours calamus *(88.77 μg/mL), respectively (Table 2).

**Table 2:** IC_50_ (μg/mL) and medicinal uses of most active plants

	**Scientific name**	**Effects and medicinal uses**	**IC**50 **(μg/mL)**
1	*Zingiber officinale*	Antiseptic, Digestive, Expectorant, Antiemetic, Antiseptic, Analgesic	48.54
2	*Laurus nobilis*	Carminative, Stomachic, Digestive	48.69
3	*Nigella sativa*	Anticancer, Analgesic, Carminative, Stomachic, Antibacterial	59.10
4	*Angelica archangelic a*	Stomachic, Antiseptic, Febrifuge, Digestive, Carminative	64.03
5	*Acours calamus*	diuretic, Antigoutal, Antiphlogistic, Stomachic, Cholagogue	88.77

Most traditional medicines are herbal ones which our information about is still insufficient. Even though medicinal plants have long been known as one of the most appropriate sources of active chemicals and their derivatives to be used as templates for designing and developing more effective compounds, preferably with less side effects, most plants having medicinal properties have not yet been thoroughly evaluated for their biological activities. With the increasing flow of medicinal plants application in the world, there is an urgent need of assessment of their complete chemical compositions (10, 11).Gastrointestinal diseases, particularly gastritis, duodenal, peptic ulcer, and gastric cancer are caused as a result of *H. pylori *infection whose habitance in the acidic medium of the stomach is highly dependence on the urease enzyme activity (11, 12). According to the literature, herbal medicines have the capability of suppressing the bacteria through inhibiting or reducing urease activity and as a result leading its ellipsis (9-12).
